# Modeling sepsis, with a special focus on large animal models of porcine peritonitis and bacteremia

**DOI:** 10.3389/fphys.2022.1094199

**Published:** 2023-01-10

**Authors:** Pavel Vintrych, Mahmoud Al-Obeidallah, Jan Horák, Jiří Chvojka, Lenka Valešová, Lukáš Nalos, Dagmar Jarkovská, Martin Matějovič, Milan Štengl

**Affiliations:** ^1^ Department of Cardiology, Faculty of Medicine in Pilsen, Charles University, Prague, Czechia; ^2^ Department of Physiology, Faculty of Medicine in Pilsen, Charles University, Prague, Czechia; ^3^ Department of Internal Medicine I, Faculty of Medicine in Pilsen, Charles University, Prague, Czechia; ^4^ Biomedical Center, Faculty of Medicine in Pilsen, Charles University, Prague, Czechia

**Keywords:** sepsis, large animal models, pig, peritonitis, bacteremia, SOFA score, cardiovascular system, immune system

## Abstract

Infectious diseases, which often result in deadly sepsis or septic shock, represent a major global health problem. For understanding the pathophysiology of sepsis and developing new treatment strategies, reliable and clinically relevant animal models of the disease are necessary. In this review, two large animal (porcine) models of sepsis induced by either peritonitis or bacteremia are introduced and their strong and weak points are discussed in the context of clinical relevance and other animal models of sepsis, with a special focus on cardiovascular and immune systems, experimental design, and monitoring. Especially for testing new therapeutic strategies, the large animal (porcine) models represent a more clinically relevant alternative to small animal models, and the findings obtained in small animal (transgenic) models should be verified in these clinically relevant large animal models before translation to the clinical level.

## 1 Introduction

Infectious diseases and their associated sepsis and septic shock remain a global health problem, with a steadily growing yearly incidence of up to 20 million cases and 5 million sepsis-related deaths worldwide (Fleischmann et al., 2016). They are among the most severe life-threatening conditions in hospital ICUs, with a mortality rate of approximately 30% (Fleischmann et al., 2016). Epidemiological studies show sepsis as one of the most crucial healthcare problems, with a significant socioeconomic impact and high financial burden for public health systems—24 billion USD is spent annually in the USA, and the situation is similar in other developed countries (Torio and Moore, 2006; Liu et al., 2014; Mellhammar et al., 2016).

**FIGURE 1:**
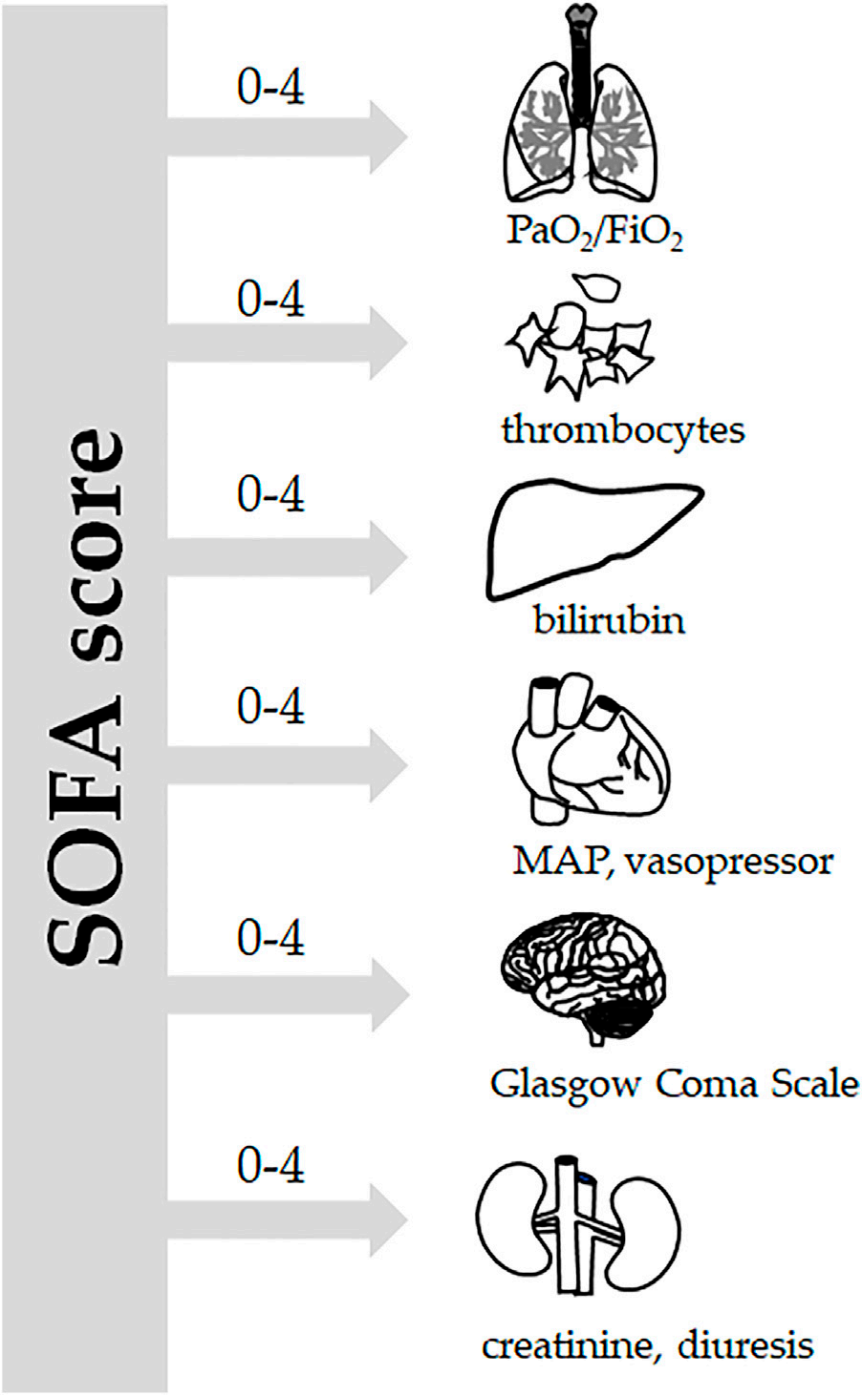
SOFA score. According to the Sepsis-3 definitions (Singer et al., 2016), sepsis is defined as life-threatening organ dysfunction caused by a dysregulated host response to infection. For clinical operationalization, organ dysfunction can be represented by an increase in the Sequential [Sepsis-related] Organ Failure Assessment (SOFA) score of two points or more. The SOFA score is based on grading six vital systems: respiration (PaO_2_/FiO_2_), coagulation (number of thrombocytes), the liver (bilirubin plasma levels), the cardiovascular system (mean arterial pressure and vasopressor support), the central nervous system (Glasgow Coma Scale), and the renal system (creatinine plasma levels, urine output), each of them scoring 0–4 points. In porcine experiments conducted under general anesthesia, the Glasgow coma scale-based neurologic component is excluded.

Sepsis is defined as life-threatening organ dysfunction caused by a dysregulated host response to infection (SEPSIS-3) (Singer et al., 2016), which corresponds to the stage of severe sepsis according to the earlier SEPSIS-1 and SEPSIS-2 definitions (Levy et al., 2003). Organ dysfunction can be identified as an acute change in the total Sequential Organ Failure Assessment (SOFA) score by >2 points. The SOFA score focuses on six vital organ systems (Figure 1): the respiratory system (PaO_2_/FiO_2_), hemostasis (number of thrombocytes), the liver (plasma levels of bilirubin), the kidneys (plasma levels of creatinine and urine volume), the central nervous system (Glasgow coma scale), and the cardiovascular system (mean arterial pressure, vasopressor support) (Singer et al., 2016; Napolitano, 2018). Septic shock is then defined as a subset of sepsis in which underlying circulatory and cellular/metabolic abnormalities are profound enough to substantially increase mortality. Patients with septic shock can be identified with a clinical construct of sepsis with persisting hypotension requiring vasopressors to maintain mean arterial pressure above 65 mmHg and having a serum lactate level above 2 mmol/L (Singer et al., 2016). For a quick bedside clinical judgement, the qSOFA (quick SOFA), which only incorporates three parameters (altered mentation, systolic blood pressure of 100 mm Hg or less, and respiratory rate of 22/min or greater) has been suggested. Although qSOFA is less robust than SOFA, it does not require laboratory tests and can be assessed quickly and repeatedly. The qSOFA criteria should be used to prompt clinicians to further investigate organ dysfunction, to initiate or escalate therapy as appropriate, and to consider referral to critical care or increase the frequency of monitoring (Singer et al., 2016; Raith et al., 2017).

Animal models allow for a detailed investigation of the pathophysiological mechanisms of the disease; however, the translation of the findings to the clinical level is often complicated. Therefore, in this review, animal models of sepsis were analyzed with an emphasis on their clinical relevance. Small and large animal models of sepsis were compared to reveal their main advantages and disadvantages with special focus on cardiovascular and immune systems. As highly clinically relevant models, two porcine models of sepsis (peritonitis- and bacteremia-induced), which are used routinely in our lab, were scrutinized in detail, including methodological aspects of the experiments.

## 2 Large vs. small animal models

Despite significant progress in our understanding of this clinical syndrome, the pathophysiology of sepsis remains unclear and there is an increasing need to improve our understanding of sepsis and develop new effective treatments for this deadly condition. Obviously, reliable and clinically relevant animal models are necessary for understanding the complex pathophysiological concepts of sepsis as well as for developing new effective treatment options. Many preclinical studies have been conducted, with promising results; however, most of them could not be successfully transferred to clinical situations. Knowledge transfer is complicated by the heterogeneity and complexity of the animal models used (Table 1). Small preclinical models are often used for modeling specific clinical situations; however, their translational potential is limited. When compared to the human phenotype of sepsis, significant differences exist in terms of their inflammatory, immune, metabolic, and hemodynamic responses to infection insult (Reade and Young, 2003; Tao et al., 2004; Alverdy et al., 2020; Wang et al., 2022). Although there are specific advantages to using genetically engineered mouse and rat models, there is an urgent need for clinically relevant large animal models that allow for an easy and reliable clinical translation. Large porcine models show, similar to humans, a hyperdynamic circulation response to infectious insult, which is in contrast to small animal (mouse, rat) models with a hypodynamic response (low cardiac output and hypotension) (Fink and Heard, 1990; Park et al., 2019). Porcine anatomy and physiology are, in general, similar to those of humans, e.g., a similar heartrate range, force–frequency relationship, and cardiac action potential duration (Meurens et al., 2012; Swindle et al., 2012). Furthermore, the similar size allows for the experimental use of regular clinical tools (probes, catheters) without modifications. When compared to other large animal model species, pigs probably represent the most plausible clinically relevant option. Since experiments with apes and dogs are currently being minimized due to ethical reasons (Balls, 2022), pigs and sheep are the most commonly used large species in sepsis research (Guillon et al., 2019). In sheep as ruminant animals, however, important differences in anatomy and physiology of the gastrointestinal tract exist, compared to omnivorous species (pig, human), which have significant metabolic consequences (Wolffram et al., 1986; Bergman 1990). On the other hand, also pigs show some species differences that may complicate clinical translation such as susceptibility to pulmonary dysfunction and acute pulmonary hypertension (Guillon et al., 2019) (Table 2).

**TABLE 1:** Overview of animal models of sepsis and septic shock.

Animal model	Procedure/induction method
**Mouse**	Cecal ligation and punction (Li et al., 2018)
	Intraperitoneal injection of *E. coli* (Uranga-Murillo et al., 2021)
	Intravenous LPS injection, intravenous or intraperitoneal injection of live *E. coli* (Zanetti et al., 1992)
	*S. aureus* mediated arthritis and sepsis (Tarkowski et al., 2001)
	Cecal slurry (Brook et al., 2019)
	Cecum ligation and dissection (Mutlak et al., 2013)
	Ascending colon stent peritonitis (Traeger et al., 2010)
	Implantation of bacteria embedded in a fibrin clot (Ghanta et al., 2021)
	Pneumonia-derived sepsis (Cai et al., 2021)
**Rat**	Cecal ligation and punction (Hua et al., 2018)
	Colon ligation and dissection (Scheiermann et al., 2009)
	Ascending colon stent peritonitis (Utiger et al., 2021)
**Hamster**	Intraperitoneal implantation of bacterial agar pellets (Whang et al., 2000)
	Intraperitoneal injection of leptospires (Haake, 2006)
	Skin fold chamber (Miranda et al., 2015)
	Subcutaneous and intradermal inoculation of leptospires (Coutinho et al., 2014)
	LPS intravenous injection (Santos et al., 2011)
**Rabbit**	Ascending colon stent peritonitis (Lu et al., 2015)
	Intravenous administration of bacteria (Zhang et al., 2020; Siddiqui et al., 2021)
	Long-term catheter-related bloodstream infections (Basas et al., 2018)
**Dog**	Sepsis *via* intestinal ischemia (Nagy et al., 1990)
	*E. coli*-infected fibrin clots implanted intraperitoneally (Solomon et al., 1994)
	Intrabronchial *S. aureus* challenge (Minneci et al., 2007)
	Peritoneal clot with *E. coli* bacteria (Natanson et al., 1988)
	Intravenous injection of live *E. coli* (Lagoa et al., 2004)
**Sheep**	Smoke inhalation injury followed by live bacteria instillation through bronchoscope (Murakami et al., 2002)
	Smoke inhalation injury followed by methicillin-resistant *S. aureus* placement into the lungs (Yaghouby et al., 2017)
	Intravenous administration of LPS (Durosier et al., 2015)
	Intravenous infusion of live bacteria (Morimatsu et al., 2012; Okazaki et al., 2021)
	Cecal ligation, perforation, and devascularization (Judges et al., 1986)
	Chorioamnionitis and fetal sepsis (Kuypers et al., 2012)
**Pig**	Fecal peritonitis, inoculating autologous feces into the abdominal cavity (Jarkovska et al., 2016)
	Bacteremia, intravenous infusion of live *P. aeruginosa* (Stengl et al., 2010a)
	Endotoxemia, infusion of *E. coli* lipopolysaccharide (Granfeldt et al., 2014)
**Rhesus monkey**	Intravenous inoculation of bacteria (Kastello and Spertzel, 1973)

**TABLE 2:** Main strengths and weaknesses of animal models of sepsis.

Animal	Strengths	Weaknesses
**Mouse**	low costs, easy handling, transgenic models for molecular insights into disease mechanisms	anatomic limits (requiring special tools), physiological and pathophysiological differences (including sepsis phenotype), limited translational potential
**Rat**	in general similar to mouse, some aspects influenced by larger size	
**Sheep**	similar size to human (use of clinical tool and interventions), repeated blood sampling, physiology and pathophysiology more similar to human good translational potential	high costs, more difficult handling, transgenic models limited, some species differences (ruminant animals with different anatomy and physiology of the gastrointestinal tract and significant metabolic consequences)
**Pig**	similar size to human (use of clinical tool and interventions), repeated blood sampling, anatomy, physiology and pathophysiology more similar to human (more than sheep), good translational potential	high costs, more difficult handling, transgenic models limited, some species differences (high susceptibility to pulmonary dysfunction), piglets (30–50 kg) are usually used (that correspond rather to pediatric patients)

## 3 The cardiovascular system in sepsis

Cardiovascular system has been extensively studied in both small and large animal models, which allows for a fair comparison with humans and a proper assessment of the possibility of clinical translation. From a practical point of view, large animal models (e.g., pigs) have an obvious advantage in that their size and macroanatomy resemble those of the human cardiovascular system. That allows for the easy use of clinical devices and tools in large animal models, including their experimental testing. Also, microstructural studies show similar characteristics for the human and porcine myocardium (Milani-Nejad and Janssen, 2014), with dominant expression of slow β–MHC (myosin heavy chain): in the porcine ventricular myocardium, it approaches 100%, with some regional variations (Stelzer et al., 2008; Locher et al., 2011); in human ventricles, expression levels of β–MHC above 90% were reported (Miyata et al., 2000; Reiser et al., 2001; Jin et al., 2017). Similar to in humans, porcine hearts express significant levels of both stiff (small) N2B and compliant (large) N2BA titin isoforms, although probably with some quantitative differences: in the porcine myocardium, a higher relative expression of the titin N2BA isoform was reported compared to in humans (Cazorla et al., 2000; Makarenko et al., 2004; Chung et al., 2011). In contrast to human and large (porcine) animal models, in the rat ventricular myocardium the fast α-MHC is dominantly expressed (>90%) (Wang et al., 2002), which has significant consequences for tension cost and kinetics (Rundell et al., 2005). Also, the expression pattern of titin isoforms in mice and rats differs from that in humans and pigs: in mice and rats, the dominant expression of N2B titin isoform with very low expression of the N2BA isoform was seen (Cazorla et al., 2000).

In general, mouse and rat hearts are adapted for very high heartrates (>600 bpm in mice), which is associated with a faster kinetics of both cardiac contraction and excitation (Milani-Nejad and Janssen, 2014). Cardiac action potential duration in mouse and rat is much shorter, and the shape is triangular without the dominant plateau phase (Odening et al., 2021). The major ionic currents responsible for repolarization are different from those in the human heart: the repolarization is mainly due to the fast and slow components of the transient outward current (I_to,f_ and I_to,s_), together with the rapidly activating, slowly inactivating delayed rectifier potassium currents (I_K, slow1_ and I_K, slow2_), whereas the main repolarization currents of human ventricular myocardium I_Kr_ (rapid delayed rectifier potassium current) and I_Ks_ (slow delayed rectifier potassium current) are functionally irrelevant (Odening et al., 2021). On the other hand, the porcine heartrate is similar (if slightly higher) to that in humans, and porcine ventricular action potentials resemble those of humans in many aspects, including the configuration with a dominant plateau phase and duration (Odening et al., 2021). Major contributing ionic currents correspond to those of humans with one exception: the transient outward current (voltage-dependent, 4-aminopyridine-sensitive I_to_) that is responsible for early repolarization in human ventricular myocytes is missing in the porcine myocardium (Li et al., 2003).

High resting heartrates in mice and rats limit the extent of additional heartrate increase, i.e., heartrate reserve, which is significantly smaller in mice and rats (∼40%) than in humans or pigs (∼150%) (Milani-Nejad and Janssen, 2014). The limited heartrate reserve, together with the rather flat force–frequency relationship in mice and rats, results in a significantly lower ability to increase cardiac output as the product of heartrate and stroke volume. The porcine cardiac reserve, on the other hand, is similar to that of humans, since the high heartrate reserve is accompanied by a positive force–frequency relationship (Vogel et al., 2003; Jarkovska et al., 2018). These differences may contribute to the differential septic response in small and large animal models, where a hypodynamic response with low cardiac output and hypotension is typical of mice and rats, whereas hyperdynamic circulation with a high cardiac output and reduced systemic vascular resistance develops in the porcine model as well as in human patients.

## 4 Immune system and sepsis modeling

The immune system plays a critical role in combating infectious stimulus and the progression of sepsis. Interspecies differences in immune mechanisms, therefore, significantly influence the outcome of the disease and potential clinical translation. A detailed analysis of portions of the porcine, mouse, and human genome associated with the immune response revealed that the porcine immune system is significantly more similar to human on the level of non-protein-coding RNA/DNA and protein-coding genes as well as proteins (Dawson and Lunney, 2018). There is a growing consensus that pigs, as monogastric omnivorous animals with an organization of the immune system very similar to that of humans, represents a suitable model for immunology, although some particularities like the inverted lymph node structure or the ileal Peyer’s patches that have no obvious anatomical equivalent in humans will require further attention (Rothkötter, 2009; Pabst, 2020). The high level of similarity between the human and porcine immune systems can also be documented by the cluster of differentiation (CD) markers: in humans, 419 proteins have been designated as CD markers, and in pigs 359 corresponding CD proteins have been identified (Dawson and Lunney, 2018). The distribution pattern of white blood cell populations in pigs is similar to that of humans, which is in contrast to mice and rats, in which the neutrophil population represents only 10%–25% of white blood cells (Fairbairn et al., 2011). Striking interspecies differences in immune effector pathways have also been described. In mice, following macrophage activation, nitric oxide (NO) is produced by calcium-independent inducible NO synthase (iNOS). NO exerts antimicrobial actions and regulates metabolic remodeling and the production of cytokines in proinflammatory macrophage (Bailey et al., 2019). It seems that these pathways are not induced in human or porcine macrophages, although there is some inconsistency in the literature about the expression of iNOS and the production of NO in human macrophages (Fairbairn et al., 2011). On the other hand, both human and porcine macrophages respond to lipopolysaccharide challenge with induction of IDO (indoleamine 2,3-dioxygenase), involved in the pathway of tryptophan metabolism, which is not the case with mouse macrophages (Kapetanovic et al., 2012). A systemic comparison of the genomic response between human inflammatory diseases (trauma, burns, and endotoxemia) and mouse models demonstrated that, although the genomic responses were highly similar in humans, they were not reproduced in mouse models (Seok et al., 2013), indicating fundamental differences in the inflammatory responses of these two species. Mice are also much more resistant (by several orders of magnitude) than humans to endotoxin shock (Warren et al., 2010). This *in vivo* discrepancy, however, was not paralleled by the *in vitro* response of macrophages in cell culture. The studies of the response of mouse and human macrophages in the microenvironment of mouse and human serum indicated that proteins in mouse serum markedly suppress the induction of proinflammatory cytokines compared to human serum (Warren et al., 2010).

Dysregulated immune response in sepsis is associated with the cytokine storm with overproduction of proinflammatory cytokines and other signaling molecules, which induce widespread endothelial dysfunction, impaired coagulation and multiple organ dysfunction (Cavaillon, 2018; Kumar, 2020). The signaling pathway of toll-like receptor 4 (TLR4) and nuclear factor κB (NF-κB) are critical for this signaling cascade. The sequence and function of porcine TLR4 is probably closer to human than mouse TLR4. Consequently, humans are highly sensitive to LPS, pigs are moderately sensitive to LPS and mice are highly resistant to LPS challenge (Vaure and Liu, 2014). Effects of cytokines are complex and often difficult to interpret. Addition of interleukin 10 (IL-10) in patients with sepsis activated the adaptive immune system by improving T-cell IFN-γ production but diminished the activity of the innate immune system by decreasing TNF-α production as well as surface expression of HLA-DR. Furthermore, in IL-10–treated septic mice an increased IFN-γ production in splenocytes was found (Mazer et al., 2019). In various murine models of sepsis associated with cytokine storm, beneficial effects of interventions aimed at reducing inflammatory mediators were shown, nevertheless the clinical translation failed (Stortz et al., 2017). In porcine models of bacteremia- and peritonitis induced sepsis, differential cytokine profile was found in pigs with and without the acute kidney injury (AKI). Despite similar septic insult and systemic hemodynamic response, only pigs with AKI showed an early increase in the plasma level of TNF-α and IL-6 (Benes et al., 2011). Similarly, in patients with pneumonia AKI was associated with higher plasma levels of both IL6 and TNF-α (Murugan et al., 2010).

Taken together, there are multiple shortcomings of mice as models for studying sepsis and inflammation, and so pigs have emerged as a clinically relevant alternative, which could allow for a better and more reliable clinical translation. Various aspects of porcine immunology and physiology have, however, not yet been analyzed in as much detail as in mice and will require further attention.

## 5 Porcine model

### 5.1 General methodological aspects

**FIGURE 2:**
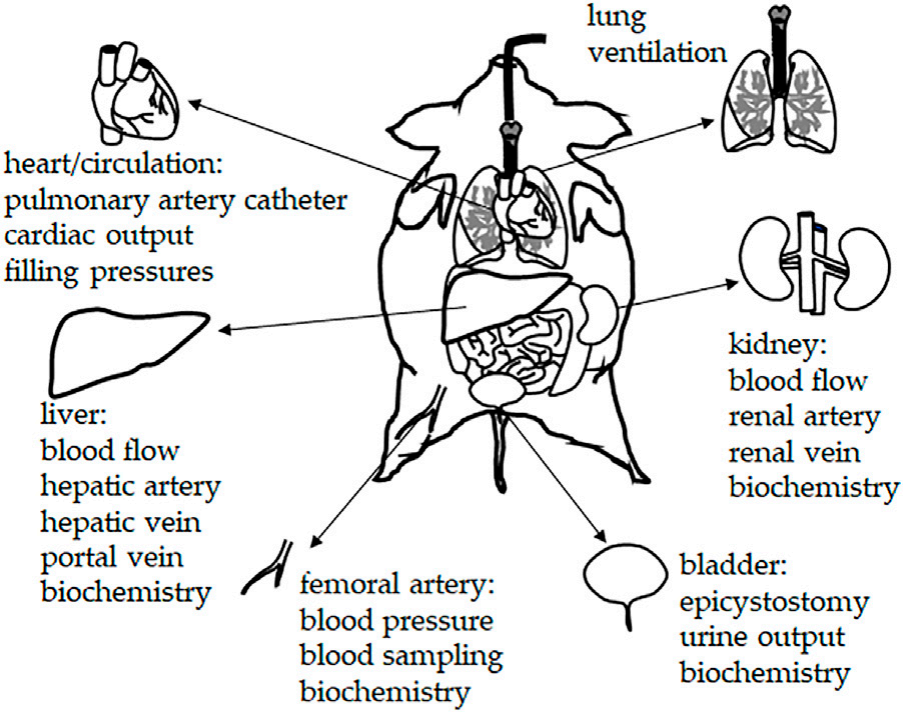
Chronic instrumentation and monitoring in porcine sepsis experiments. The experimental setting may include: central venous catheter for drug and fluid infusion (inserted through the left jugular vein); balloon-tipped thermodilution pulmonary artery catheter (placed *via* the right jugular vein); femoral arterial catheter for blood pressure recording and blood sampling; fiberoptic catheter for thermal-dye double-indicator dilution measurements; ultrasound transit time flow probes (e.g., around the portal vein, the common hepatic artery, and the left renal artery); catheters in the portal, renal, and hepatic veins; laser Doppler flowmetry for monitoring ileal mucosal and renal cortex microcirculation; cystostomy catheter for urine collection (percutaneous insertion under ultrasound guidance).

**FIGURE 3:**
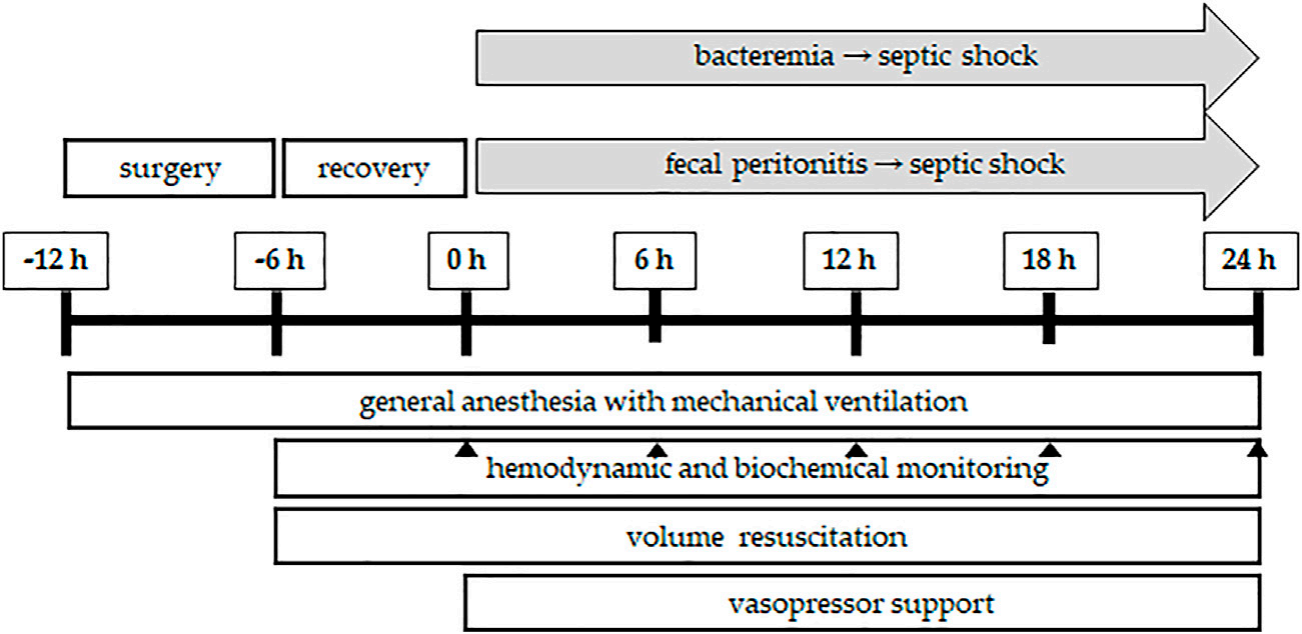
Flowchart of porcine sepsis experiments. After induction of general anesthesia and mechanical ventilation, the experiment starts with a surgical preparatory phase, during which access to various vital organ systems is secured and chronic instrumentation devices (see Figure 2) are installed. A postsurgical stabilization period of 6 h is allowed before baseline measurements are obtained (time point: 0 h). Afterwards, sepsis is induced either by bacteremia (continuous central venous infusion of the bacteria of interest, e.g., live *P. aeruginosa*) or fecal peritonitis (inoculating autologous feces suspended in saline into the abdominal cavity through the drainage tubes). Sepsis progression is followed for 24 h, during which, in a usual setting, irreversible septic shock develops. Throughout the experiment, general anesthesia and mechanical ventilation are maintained with proper volume resuscitation to maintain cardiac filling pressures and administration of vasopressor support, if needed, to maintain the mean blood pressure above 65 mmHg. Continuous hemodynamic monitoring is accompanied by blood and urine sampling for biochemical analyses at the time points of interest (usually 6, 12, 18, and 24 h from the induction of sepsis). At the end of the *in vivo* experiment, the animals are euthanized by anesthetic overdose and excision of the heart. The total duration of the *in vivo* porcine sepsis experiment is 36 h and it is followed by *in vitro* analysis of tissues, cells, and subcellular organelles.

An important advantage of large (porcine) animal models is that extensive monitoring of vital functions is plausible (Figures 2, 3). A standard experimental setting may include: a central venous catheter for drug and fluid infusion (inserted through the left jugular vein); a balloon-tipped thermodilution pulmonary artery catheter (placed *via* the right jugular vein); a femoral arterial catheter for blood pressure recording and blood sampling; a fiberoptic catheter for thermal-dye double-indicator dilution measurements; ultrasound transit time flow probes (e.g., around the portal vein, the common hepatic artery, and the left renal artery); catheters in the portal, renal, and hepatic veins; laser Doppler flowmetry for monitoring ileal mucosal and renal cortex microcirculation; and a cystostomy catheter for urine collection (percutaneous insertion under ultrasound guidance). In case hemofiltration techniques are investigated, a dialysis catheter (e.g., 14-French double-lumen) may be inserted into the right femoral vein and serve for hemofiltration access (Stengl et al., 2010b; Jarkovska et al., 2016; Jarkovska et al., 2018; Al-Obeidallah et al., 2021). Such extensive monitoring allows for a determination of the modified SOFA score according to the Third International Consensus definitions of sepsis and septic shock (Singer et al., 2016) with the exclusion of the Glasgow coma scale-based neurologic component due to the use of general anesthesia. In general, using the (modified) SOFA score in (large) animal studies allows for comparison and a better standardization of experimental conditions and results. In our opinion, a wide implementation of the SOFA score into animal sepsis research will facilitate the clinical translation of experimental results.

Bacteremia can be induced by continuous central venous infusion of bacteria of interest. In our earlier study, live *P. aeruginosa* (strain O1, isolated from a patient with suppurative otitis, 1 x 109 colony-forming units/mL determined by serial dilution and colony counts) was used (Stengl et al., 2010a). The infusion rate was titrated throughout the experiment to result in moderate pulmonary hypertension (mean pulmonary artery pressure 35–40 mmHg). Peritonitis can be induced by inoculating autologous feces (0.5–2 g·kg-1, suspended in 200 mL saline) into the abdominal cavity through drainage tubes placed through the abdominal wall (Jarkovska et al., 2016). In our experimental setting, inoculation of high doses of feces (>1 g·kg-1) is usually necessary for reaching the septic shock stage within 24 h. On the other hand, inoculation of low doses of feces (0.5 g·kg-1) usually only results into development of sepsis but not septic shock (Al-Obeidallah et al., 2021). In peritonitis model, a significant variability in the host response is often encountered. As a potential source of this heterogeneity, fecal microbiome was analyzed and interestingly, a significant difference in bacterial composition was associated with the season (winter vs. spring/autumn). It seems, that the seasonal diversity of the microbiome composition could significantly influence outcomes of this experimental model of sepsis (Chalupova et al., 2022).

Regardless of the infectious insult, general anesthesia is maintained throughout the experiment through a combination of continuous intravenous thiopental and fentanyl infusions. Animals are mechanically ventilated and receive appropriate volume resuscitation. Continuous i.v. norepinephrine is administered if needed to maintain a mean blood pressure of 70–75 mmHg.

### 5.2 Peritonitis vs. bacteremia in a porcine model

**FIGURE 4:**
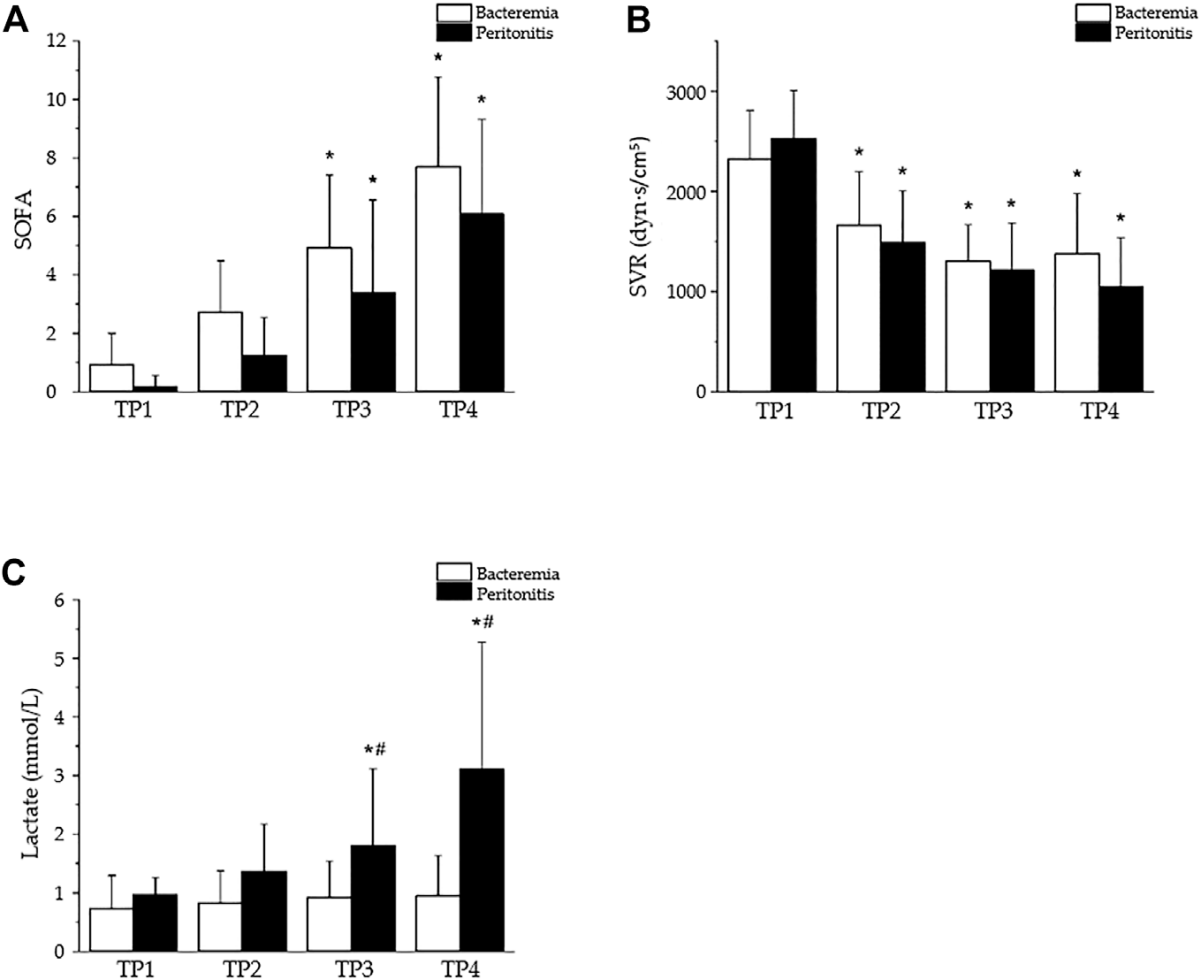
Selected parameters of sepsis progression in a porcine model of bacteremia- or peritonitis-induced sepsis. The bacteremia was induced by continuous central venous infusion of live *P. aeruginosa* (strain O1 isolated from a patient with suppurative otitis, 1 x 109 colony-forming units/mL) (Stengl et al., 2010a); the peritonitis was induced by inoculating autologous feces (0.5–2 g·kg-1, suspended in 200 mL saline) into the abdominal cavity (Jarkovska et al., 2016). Peritonitis, n = 13; bacteremia, n = 14; based on our previous publications (Stengl et al., 2008; 2010a; 2010b). TP1–4: time points 0, 12, 18, and 24 h after sepsis induction. Data are presented as the mean ± SD; *p < 0.05, vs. TP1; #*p* < 0.05, bacteremia vs. peritonitis. **(A)** Similar progression of sepsis in pigs with bacteremia and peritonitis documented by rising SOFA scores. **(B)** Similar hemodynamic alterations in both peritonitis- and bacteremia-induced sepsis documented by reduced systemic vascular resistance (SVR). **(C)** Plasma levels of lactate as example of differential response in peritonitis- and bacteremia-induced sepsis.

With appropriate dosing, both peritonitis and bacteremia can lead to irreversible septic shock within 24 h, with similar general dynamics of sepsis progression (Figure 4A) and significant peripheral vasodilation (Figure 4B). Significant differences, however, developed in terms of the plasma levels of lactate, which were significantly increased in peritonitis but not bacteremia (Figure 4C). In contrast to the traditional view of hyperlactatemia as a product of oxygen debt and anaerobic metabolism, the current interpretation of lactate metabolism is more complex (Brooks, 2020). Hyperlactatemia develops during sepsis and septic shock, probably as a result of the dynamic balance between lactate production and clearance in various tissues and cells (Gibot, 2012). Although, in some studies, impaired lactate clearance was identified (Levraut et al., 1998), lactate overproduction is probably more important. Increased lactate formation was found in the skeletal muscles of patients with septic shock (Levy et al., 2005) as a result of exaggerated aerobic glycolysis through Na+/K + ATPase stimulation. In mild endotoxemia, however, skeletal muscle was unlikely to be a major contributor to increased lactate production (Michaeli et al., 2012). Increased glucose consumption and lactate production mediated through the MEK/ERK signaling cascade was also shown in LPS-activated mouse macrophages, suggesting a tight cross-talk between inflammatory signal transduction and metabolic networks (Través et al., 2012). Obviously, the precise origin of lactate in sepsis may differ in different clinical conditions; it is probably multifactorial and will require further elucidation.

## 6 Concluding remarks

Three broad categories of experimental models of sepsis include locally induced sepsis (e.g., peritonitis), endotoxemia, and the intravenous administration of a viable pathogen (intermittent or continuous bacteremia). Each model has its strengths and limitations. Intravenous application of single live bacteria (e.g., *Pseudomonas*, *E. coli*) is technically simple and reproduces many pathophysiological features of human sepsis. This type of modeling allows for a tightly regulated load of bacterial exposure, and, therefore, severity of insult. Clinically, it replicates conditions such as acute endocarditis or overwhelming infections with Pneumococcus or Meningococcus. In addition, the specific host response to Gram-positive or Gram-negative bacterial challenge can be investigated. On the other hand, the intravenous application of live bacteria leads to a rapid immune-inflammatory and hemodynamic response, usually not seen in sepsis induced by an infectious focus. Hence, more complex, and perhaps more clinically relevant models include locally induced sepsis such as peritonitis. These models of polymicrobial sepsis allow for more protracted immune-inflammatory, metabolic, and hemodynamic alterations to be observed in a clinical setting. Autologous fecal material challenges the animals with its individual gut flora. However, marked intra- and interindividual variations in gut microbial diversity might predispose animals to significant heterogeneity in their host response. Another problem is the difficulty of controlling the quantity of bacteria introduced into the peritoneum.

In a wider context, there is a growing interest in a more tailored approach to sepsis treatment. This approach would require identification of subgroups of patients expressing phenotypic or even endotypic similarity that reflect unique pathophysiology. Indeed, many recent clinical studies described different sepsis phenopytes allowing to identify high-risk patients and those with high probability of responding well to targeted treatments (Seymour et al., 2019; Stanski and Wong, 2020; DeMerle et al., 2021). Unfortunately, to the best of our knowledge, there are no clearly defined large animal model of sepsis phenotypes, in which to explore underlying pathophysiology or to test novel therapeutic targets. Nevertheless, we have recently demonstrated the associations of outcome with clinically relevant phenotypes (Al-Obeidallah et al., 2021). In that study, SOFA score, hemodynamical parameters and body temperature were shown to significantly and early discriminate between sepsis and septic shock in a clinically relevant porcine model. This subgroup of animals nicely corresponds to a group A sub-phenotype characterized by hyperthermia, tachycardia, hypotension and significantly higher odds of mortality as shown in a recent large clinical trial (Bhavani et al., 2022). Our data suggest, that sepsis sub-phenotyping based on vital sign and pattern of organ dysfunction trajectory is feasible even in pre-clinical research of sepsis. Clearly, further studies exploring the correlation between these phenotypes and mechanism-based sepsis endotyping are necessary.

It may be concluded that large animal (porcine) models represent a more clinically relevant alternative to small animal (mouse, rat) models, especially for testing novel therapeutic strategies with not so clear cellular and molecular mechanisms. In a number of studies, porcine models of sepsis have shown a high level of correspondence with clinical situations and human disease progression, thus allowing for the easy and reliable translation of experimental findings to a clinical setting. On the other hand, small animal models, especially transgenic models, allow researchers to obtain mechanistic proof-of-principle insights into the molecular mechanisms of disease in a much faster and more cost-effective way. Obviously, the relationship between small and large models is not competitive but complementary, and the optimal translation should include both steps, with the verification of small animal model findings in large animal models.
